# Prognostic Values of the Gray-to-White Matter Ratio on Brain Computed Tomography Images for Neurological Outcomes after Cardiac Arrest: A Meta-Analysis

**DOI:** 10.1155/2020/7949516

**Published:** 2020-11-03

**Authors:** Wen Jie Wang, Jie Cui, Guang Wei Lv, Shun Yi Feng, Yong Zhao, Su Li Zhang, Yong Li

**Affiliations:** Emergency Department, Cangzhou Central Hospital, No. 16 Xinhua Road, Yunhe Qu, Cangzhou 061000, China

## Abstract

**Materials and Methods:**

The PubMed, ScienceDirect, Web of Science, and China National Knowledge Infrastructure databases were searched for all relevant articles published before March 31, 2020, without any language restrictions. The pooled odds ratios (ORs) and 95% confidence intervals (CIs) were calculated with a random-effects model using Stata 14.0 software.

**Result:**

A total of 24 eligible studies with 2812 CA patients were recruited in the meta-analysis. The pooled result showed that decreased GWR was correlated with poor neurological outcomes after CA (OR = 11.28, 95% CI: 6.29–20.21, and *P* < 0.001) with moderate heterogeneity (*I*^2^ = 71.5%, *P* < 0.001). The pooled sensitivity and specificity were 0.58 (95% CI: 0.47–0.68) and 0.95 (95% CI: 0.87–0.98), respectively. The area under the curve (AUC) of GWR was 0.84 (95% CI: 0.80–0.87). Compared with GWR (cerebrum) and GWR (average), GWR using the basal ganglion level of brain CT had the highest AUC of 0.87 (0.84–0.90). Subgroup analysis indicated that heterogeneity may be derived from the time of CT measurement, preset specificity, targeted temperature management, or proportion of cardiac etiology. Sensitivity analysis indicated that the result was stable, and Deeks' plot showed no possible publication bias (*P* = 0 .64).

**Conclusion:**

Current research suggests that GWR, especially using the basal ganglion level of brain CT, is a useful parameter for determining neurological outcomes after CA.

## 1. Introduction

Cardiac arrest (CA) is a growing public health issue carrying an enormous global burden of morbidity, and out-of-hospital CA accounts for approximately 10% of individuals who survive to hospital discharge [1]. Unfortunately, the incidence of neurological sequelae among survivors of CA remains as high as 70% despite advances in post-CA care [2, 3]. Neurological sequelae have a significantly negative impact on the community in terms of life years lost and healthcare costs in survivors and the emotional burden of family members. Early clinical predictors in survivors of CA are important for counseling families and making management decisions.

Some signs on brain computed tomography (CT) that have been associated with ischemic cerebral insult include a loss of boundary between the gray matter (GM) and the white matter (WM). Several studies [4–27] indicate that a loss of differentiation between GM and WM, reflected as decreased gray-to-white matter ratio (GWR) on brain CT, predicts a poor outcome in CA patients. However, in the absence of large and comprehensive diagnostic studies, the prognostic role of GWR in survivors of CA is still controversial. Therefore, we performed a meta-analysis to evaluate the prognostic value of GWR for neurological outcomes after CA.

## 2. Methods

This meta-analysis was carried out according to the Preferred Reporting Items for Systematic Reviews and Meta-Analyses statement [28]. We registered our systematic review prospectively in PROSPERO (CRD42020182066). As this study was a review of published literature, the approval of an ethics committee and consent of patients were not required.

### 2.1. Literature Search

The PubMed, ScienceDirect, Web of Science, and China National Knowledge Infrastructure databases were searched for all relevant articles published before March 31, 2020, without any language restrictions. The search terms included “gray-to-white ratio” and “cardiac arrest.” Moreover, the references from the retrieved studies were also reviewed by manual search to identify any new eligible studies.

Studies were included if they satisfied the following criteria: the full-text publication evaluated the association of GWR on brain CT for neurological outcomes, and sufficient information should be available to evaluate odds ratios (ORs) with 95% confidence intervals (CIs). Letters, comments, editorials, case reports, communications, and duplicated studies were excluded.

### 2.2. Data Extraction and Quality Assessment

The data extraction of the present study was independently performed by two reviewers. The extracted data included the first names of the author, country, study design, sample size, time of brain CT measurement, targeted temperature management (TTM), area under the curve (AUC), cutoff value, GWR value, proportion of cardiac etiology, and outcomes for each study. The caudate nucleus (CN), putamen (PU), posterior limb of the internal capsule (PLIC), and corpus callosum (CC) were measured for the basal ganglia, and the gray and white matter from the medial cortex was measured at the centrum semiovale (MC1, MW1) and high cortical level (MC2, MW2). The GWR for the basal ganglia (GWR‐BG = (CN + PU)/(PLIC + CC)), GWR for the cerebrum (GWR‐CE = (MC1 + MC2)/(MW1 + MW2)), and the average of the two (GWR‐AV = (GWR‐BG + GWR‐CE)/2) were calculated as previously described [4]. The simplified GWR estimation method (GWR‐SI = PU/PLIC, CN/PLIC, or PU/CC) was calculated as previously described [7].

Quality Assessment of Diagnostic Accuracy Studies 2 was used to assess the reporting quality of the included original studies [29]. If there was disagreement, a consensus was reached by a third reviewer.

### 2.3. Statistical Analysis

Statistical analyses will be performed using Stata/MP 14.0 (StataCorp, College Station, TX, USA). The associations between GWR and neurological outcomes after CA were estimated on the basis of the pooled ORs and 95% CIs. Heterogeneity was assessed by using the *I*^2^ statistic where *P* < 0.1 and/or *I*^2^ > 50% indicated heterogeneity between the data, and the combined analysis was performed using a random-effects model. Otherwise, a fixed-effects model was used. Publication bias was formally assessed using Deeks' plot if more than 10 qualified studies are included in our study. Additionally, we used sensitivity analysis to evaluate the stabilization of the study. For all statistical analyses, *P* < 0.05 was considered to indicate statistical significance, and all tests were two-sided.

## 3. Results

### 3.1. Literature Search

**Figure 1:**
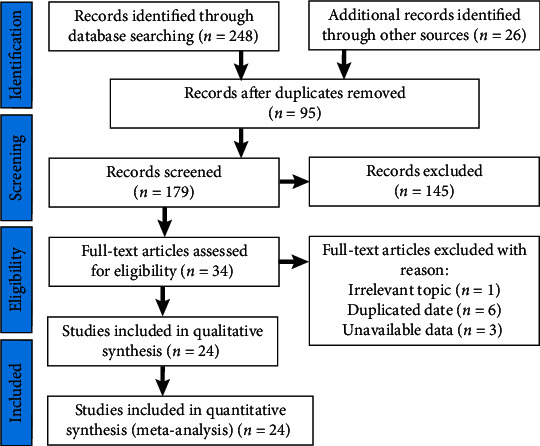
Flow diagram showed the selection process of meta-analysis.

The general characteristics of the included studies are summarized in Figure 1. Initially, 274 publications were retrieved by the mentioned search strategy, of which 95 duplicated studies were excluded. Of the remaining 179 studies, 155 were excluded for being reviews, comments, or abstracts and unrelated to the topic. After screening the title, abstract, and full text, 24 studies [4–27] that involved a total of 2812 patients were eligible for inclusion.

### 3.2. Characteristics of the Included Studies

The characteristics of the included studies are summarized in Table 1. Of the 24 studies [4–27], 22 were retrospective cohort studies [4–19, 21, 23–27], and two were prospective in nature [20, 22]. The number of included patients per study ranged from 25 to 346, with an average of 115. The reported cutoff of GWR in the included studies varied between 1.07 and 1.26. The AUC of GWR in different studies varied between 0.650 and 0.947.

### 3.3. Quality Assessment

**Figure 2:**
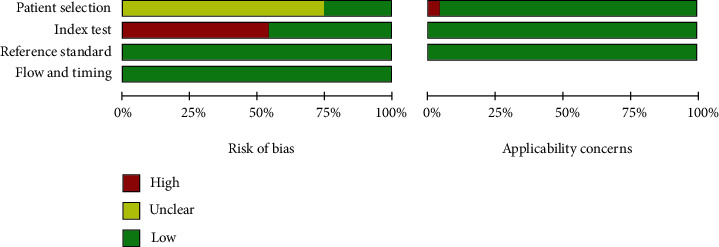
Flow diagram of studies' selection and quality assessment of the included articles.

The patient selection risk of bias domain in 18 studies [4, 5, 7, 9–11, 13–21, 23, 24, 26] was labeled as unclear risk because the authors did not indicate whether patients were recruited consecutively, and the index text risk of bias domain in 13 studies [4, 6, 8, 11, 14, 16, 17, 19–21, 24, 26, 27] was labeled high risk because the authors did not have a preset specificity. In addition, the patient selection risk of bias domain in one study [25] was labeled as unclear risk because patients with a normal brain CT scan served as controls (Figure 2 and additional Figure 1).

### 3.4. Diagnostic Performance

**Figure 3:**
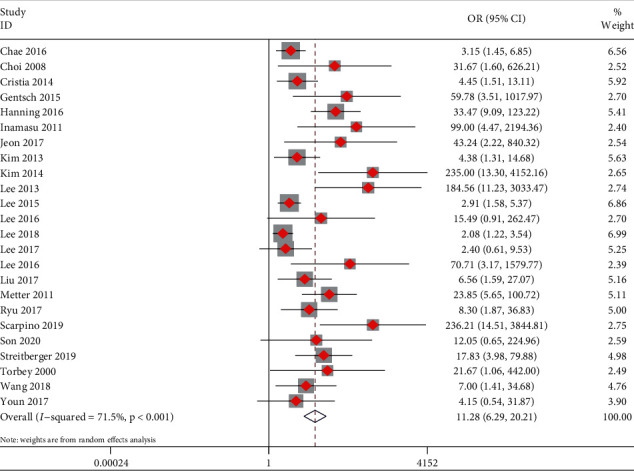
Forest plots for the meta-analysis of the prognostic value of the gray-to-white matter ratio for neurological outcomes after cardiac arrest.

**Figure 4:**
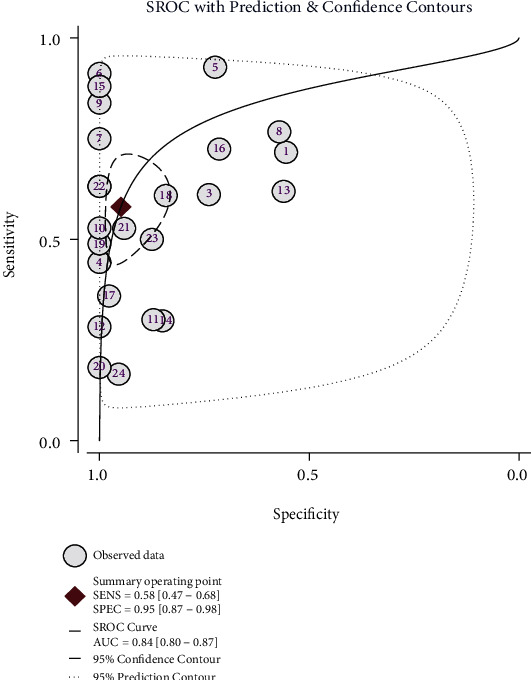
Summary ROC curve for estimating the testing accuracy of the gray-to-white matter ratio for neurological outcomes after cardiac arrest.

As shown in Figure 3, the pooled results demonstrated that decreased GWR was associated with poor prognosis in post-CA patients (OR = 11.28, 95% CI: 6.29–20.21, and *P* < 0.001; *I*^2^ = 71.5%, *P* < 0.001). As shown in Figure 4, the pooled sensitivity and specificity were 0.58 (95% CI: 0.47–0.68) and 0.95 (95% CI: 0.87–0.98), respectively. The positive likelihood ratio was 11.4 (95% CI: 4.60–28.40), the negative likelihood ratio was 0.44 (95% CI: 0.35–0.56), the diagnostic OR was 26 (95% CI: 10–69), and the AUC was 0.84 (95% CI: 0.80–0.87).

Eight studies [7, 11, 14, 15, 18, 19, 21, 26] compared the prognostic performance of GWR-BG, GWR-CE, and GWR-AV. The pooled results (Table 2) demonstrated that GWR using the basal ganglion level of brain CT has the highest AUC of 0.87 (0.84–0.90).

### 3.5. Subgroup Analysis

Due to heterogeneity, subgroup analysis was conducted on the basis of the preset specificity (preset specificity = 1 or nonpreset specificity), sample size (<100 or ≥100), study design (retrospective or prospective), country (Asia or non-Asia), time of CT measurement (≤24 h or >24 h), TTM (all or not all), cutoff (≤1.18 or >1.18), cardiac etiology (≤50% or >50%), and row of CT (16 rows or 64 rows) for a subsequent investigation of potential heterogeneity. We found that nonpreset specificity, time of CT measurement, TTM, or proportion of cardiac etiology may cause heterogeneity but did not affect the final conclusion (Table 3).

### 3.6. Sensitivity Analysis and Publication Bias Assessment

**Figure 5:**
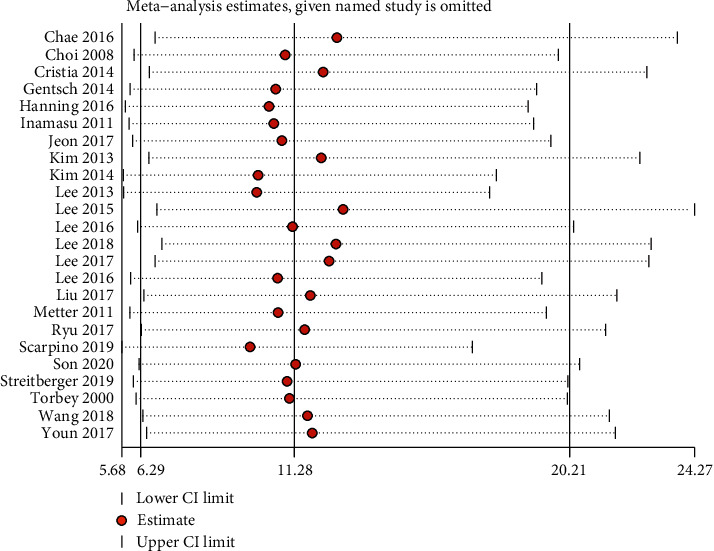
Sensitivity analysis to examine the influence of each individual study on the overall results.

**Figure 6:**
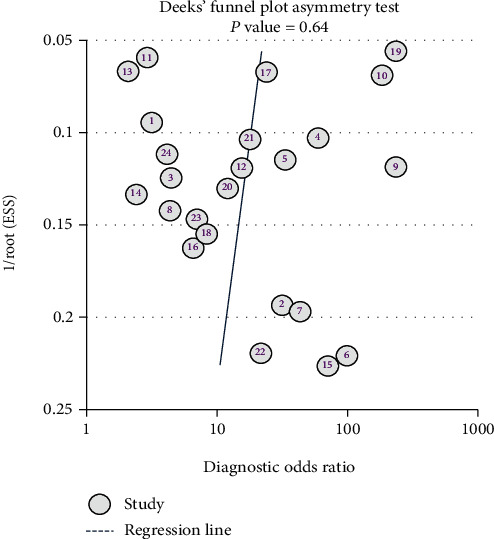
Deeks' plot for the publication bias test.

Sensitivity analysis did not find any single study that had an impact on the total pooled effect, indicating that the result was stable (Figure 5). Publication bias was examined using Deeks' plot asymmetry test, and the funnel plot did not reveal significant publication bias (*P* = 0 .64; Figure 6).

## 4. Discussion

In this systematic review and meta-analysis, we evaluated the diagnostic accuracy of GWR for neurological outcomes after CA by analyzing the current clinical evidence. Our principal findings were as follows: the predictive ability of GWR for a poor neurological outcome assessed using the AUC was 0.84 (95% CI: 0.80–0.87). Furthermore, GWR using the basal ganglion level of brain CT had the highest AUC of 0.87 (0.84–0.90).

A previous meta-analysis by Lopez Soto et al. [30] showed that a decreased GWR brain CT is useful for predicting poor neurological outcomes with a sensitivity of 0.44 and specificity of 0.97; this finding is consistent with our results. However, we comprehensively searched the PubMed, ScienceDirect, Web of Science, and China National Knowledge Infrastructure databases, and four additional studies were considered in the present meta-analysis. In addition, we compared the prognostic performance of GWR measurement methods, including GWR-BG, GWR-CE, and GWR-AV, and discovered that the GWR using GWR-BG had the highest performance.

The AUC of GWR-BG was higher than those of GWR-CE and GWR-AV for the following reasons. The relative attenuations of GM and WM throughout various regions of the brain show discrepancy. Gentsch et al. [7] speculated that the basal ganglia are more severely damaged by hypoxia due to their high metabolic activity and their location within the boundary zones of perfusion or measurement of Hounsfield units in the cortical GM is less reliable due to partial volume effects [31].

There is currently no consensus on a distinct GWR cutoff value that may predict a poor outcome with high specificity. The reported cutoff of GWR in the 24 included studies varied between 1.07 and 1.26. In addition, the AUC of GWR showed great discrepancy in different studies and varied between 0.650 and 0.947. This discrepancy may be associated with the following reasons. First, Morimoto et al. [32] reported that cerebral edema is more common after CA of respiratory etiology due to the development of metabolic acidosis (possibly lactic acidosis) induced by hypoxia. Similarly, Lee et al. [15] suggested that noncardiac etiology is associated with a more severe brain edema than cardiac etiology. A change in GWR values is associated with the water content of the brain tissue; therefore, patients with respiratory or noncardiac etiology CA may have lower GWR in the initial brain CT. Second, the timing for brain CT scans was not standardized.

Subgroup analysis indicated that nonpreset specificity, time of CT measurement, TTM, and proportion of cardiac etiology may cause heterogeneity. Although conclusions based on pooled estimates in subgroups are consistent, clinicians should consider these heterogeneity factors in their clinical practice. Several studies [5, 7, 9, 10, 12, 13, 15, 18, 22, 23, 25] have determined cutoff values with 100% specificity for predicting poor neurological outcomes to identify patients with a minimal chance of achieving a good neurological outcome, which could partly affect the difficulty of deriving conclusions based on pooled estimates. Survival with a favorable neurological outcome may be possible in patients with hypothermia despite a severe early injury, which influences the predictive value GWR for the neurological outcome. Thus, the reevaluation of the predictive value of GWR is necessary when patients receive hypothermia therapy. Metter et al. [20] proved that the time from arrest to CT is not related to GWR or attenuation values although cerebral edema after CA evolves over time. Notably, brain CT scans performed during the first 2 h after ROSC may not provide sufficient time for the formation of cerebral edema and GWR within this time window and are not a good outcome predictor [15, 33]. Noncardiac etiology, instead of cardiac etiology, may lead to severe brain injury with an eventual poor clinical outcome; GWR is more helpful in predicting neurological outcomes in CA patients with noncardiac etiologies than with cardiac etiologies [15]. However, we failed to perform a subgroup analysis on the basis of the slice thickness of brain CT scanners because only one study used 2.5–4.8 mm slice thickness; the others used 5 mm slice thickness. However, Oh et al. [34] obtained a significant variance in Hounsfield units when different CT scanners were used but observed only minor differences in GWR values. Hanning et al. [8] utilized coregistration with an atlas to calculate the average GWR, but the obtained value differs from the average GWR calculated through the manual placement of a few regions of interest. This condition indicates that the computing method, whether automated or manual, needs to be considered when assessing the predictive value of GWR.

Several limitations should be carefully considered in the present study. First, our analysis was based mainly on findings from retrospective studies, which might contain a higher number of confounding factors than prospective studies. Second, the studies involved in this meta-analysis had varying cutoff values. Third, the included studies exhibited significant heterogeneity, which may have reduced the reliability of the analysis. Fourth, neuroprognostication studies are potentially susceptible to a self-fulfilling prophecy because the investigated prognostic parameters may affect the withdrawal of life-sustaining therapy. Lastly, the timing of brain CT is critical to the sensitivity of GWR to poor outcome prediction, and some included studies did not perform CT at optimal time points (within 6 h and usually around 2 h) [17]. Further research is needed to establish the optimal timing of brain CT measurement.

## 5. Conclusions

Brain CT is simple, cost-effective, and easily implemented after CA. Stratification analysis based on CT scan obtained at different time points after CA, etiology induced by CA, and therapeutic hypothermia is required to understand the pattern of GWR and neurological outcomes.

## Figures and Tables

**Table 1:** Study characteristics of included studies.

First author	Publication year	Country	Time of CT measurement	Study design	Cardiac etiology (%)	Method of GWR measurement	Sample size	CT scanner (manufacturers/row/slice thickness)	GWR calculation	TTM (%)	AUC	Cutoff	GWR value	
													Good outcome	Poor outcome
Chae et al. [4]	2016	Korea	Within 6 h after ROSC	R	56%	GWR-AV	119	GE/NA/5 mm	Manual	100%	0.65	1.24	1.23 (0.05)	1.19 (0.08)
Choi et al. [5]	2008	Korea	Within 24 h after resuscitation	R	46%	GWR-BG	28	GE/NA/5 mm	Manual	NA	0.947	1.22	1.28 (0.01)	1.19 (0.01)
Cristia et al. [6]	2014	USA	Within 24 h after ROSC	R	NA	GWR-AV	77	GE/16 or 64 rows/5 mm	Manual	70%	0.64	1.20	NA	NA
Gentsch et al. [7]	2015	Germany	Within 7 days after arrest	R	53%	GWR-SI	98	GE/16 rows/5 mm	Manual	100%	0.810	1.11	NA	NA
Hanning et al. [8]	2016	Germany	Within 24 h after arrest	R	39%	GWR-SI	84	NA	Automated	NA	0.860	1.084	1.10 (1.08-1.12)	1.06 (1.05-1.08)
Inamasu et al. [9]	2011	Japan	Within 2 h of arrival	R	0%	GWR-SI	39	GE/16 rows/5 mm	Manual	NA	NA	NA	NA	NA
Jeon et al. [10]	2017	Korea	Within 6 h after arrest	R	49%	GWR-AV	39	Philips/64 rows/NA	Manual	0%	0.891	1.21	1.27 (1.24-1.31)	1.17 (1.14-1.21)
Kim et al. [11]	2013	Korea	Within 1 h after resuscitation	R	69%	GWR-AV	51	GE/64 rows/5 mm	Manual	100%	0.747	1.24	1.26 ± .07	1.21 ± .06
Kim et al. [12]	2014	Korea	Within 24 h after ROSC	R	38%	GWR-AV	91	NA	Manual	44%	0.922	1.23	1.29 ± 0.05	1.17 ± 0.07
Lee et al. [13]	2013	Korea	Within 3.5 h after ROSC	R	76%	GWR-SI	224	NA	Manual	100%	0.864	1.17	1.25 (1.21-1.32)	1.17 (1.13-1.21)
Lee et al. [14]	2015	Korea	Within 24 h after ROSC	R	100%	GWR-AV	283	NA	Manual	100%	0.591	1.26	1.318 (1.285-1.368)	1.302 (1.257-1.350)
Lee et al. [15]	2016	Korea	Within 24 h after ROSC	R	0%	GWR-AV	164	NA	Manual	100%	0.638	1.22	NA	NA
Lee et al. [16]	2018	Korea	Within 24 h after resuscitation	R	52%	GWR-SI	258	NA	Manual	100%	0.60	1.14	1.15 (1.11-1.22)	1.13 (1.07-1.19)
Lee et al. [17]	2017	Korea	Within 6 h after arrest	R	55%	GWR-AV	67	Siemens/16 rows/5 mm	Manual	100%	NA	1.13	1.20 ± 0.07	1.18 ± 0.09
Lee et al. [18]	2016	Korea	Within 1 h after ECMO pump	R	NA	GWR-BG	30	Siemens/64 rows/5 mm	Manual	100%	0.872	1.24	1.28 (1.24-1.40)	1.17 (1.12-1.24)
Liu et al. [19]	2017	China	Within 5 days after ROSC	R	60%	GWR-BG	43	NA	Manual	100%	0.756	1.13	1.163 ± 0.818	1.064 ± 0.103
Metter et al. [20]	2011	USA	Within 24 h after arrest	P	NA	GWR-AV	240	GE/NA/5 mm	Manual	70%	0.72	1.20	1.26 (1.24-1.30)	1.22 (1.16-1.27)
Ryu et al. [21]	2017	Korea	Within 48 h after ECPR	R	83%	GWR-BG	42	GE/64 rows/5 mm	Manual	38%	0.792	1.23	1.31 (1.25-1.37)	1.21 (1.11-1.28)
Scarpino et al. [22]	2019	Italy	Within 24 h after arrest	P	NA	GWR-BG	346	NA	Manual	40%	0.87	1.21	NA	NA
Son et al. [23]	2020	Korea	Within 6 h after ROSC	R	30%	GWR-AV	58	Siemens/64 rows/5 mm	Manual	0%	0.719	1.07	1.24 (1.19-1.29)	1.16 (1.11-1.24)
Streitberger et al. [24]	2019	Germany	24 h-10 days after arrest	R	46%	GWR-AV	108	GE/NA/5 mm	Manual	100%	0.80	1.13	NA	NA
Torbey et al. [25]	2000	USA	Within 24 h after arrest	R	NA	GWR-AV	25	GE/NA/5 mm	Manual	NA	NA	1.18	NA	NA
Wang et al. [26]	2018	China	Within 72 h after resuscitation	R	22%	GWR-BG	58	GE/16 rows/5 mm	Manual	100%	0.698	1.18	1.24 (1.20-1.32)	1.19 (1.11-1.25)
Youn et al. [27]	2017	USA	Within 24 h after arrest	R	NA	GWR-AV	240	GE/NA/5 mm	Manual	91%	0.726	1.10	1.27 ± 0.09	1.20 ± 0.11

AUC: area under the curve; AV: average; BG: basal ganglia; ECMO: extracorporeal membrane oxygenation; ECPR: extracorporeal cardiopulmonary resuscitation; GWR: gray-to-white ratio; P: prospective; R: retrospective; SI: simplified estimation method; TTM: targeted temperature management.

**Table 2:** Prognostic performance of GWR using different regions of the brain.

	OR (95% CI)	*P*	*I* ^2^ (*P* value)	Sensibility	Specificity	AUC
GWR-BG	15.77 (6.57–37.71)	<0.001	0% (0.690)	0.32 (0.12–0.60)	1.0 (0.13–1.0)	0.87 (0.84–0.90)
GWR-CE	11.06 (4.68–26.16)	<0.001	0% (0.947)	0.27 (0.14–0.45)	1.0 (0.24–1.00)	0.72 (0.68–0.76)
GWR-AV	15.92 (6.88–36.83)	<0.001	59.6% (0.722)	0.35 (0.18–0.58)	1.0 (0.14–1.00)	0.79 (0.75–0.82)

AUC: area under the curve; AV: average; BG: basal ganglia; CE: cerebrum; CI: confidence interval; GWR: gray-to-white ratio; OR: odds ratio.

**Table 3:** Subgroup analysis.

	*N*	OR (95% CI)	*P*	*I* ^2^ (*P* value)
Specificity				
1	11	58.90 (24.40–142.22)	<0.001	0.0% (0.852)
Not 1	13	5.44 (3.33–8.88)	<0.001	60.5% (0.002)
Sample size				
<100	15	12.08 (6.32–23.06)	<0.001	42.6% (0.041)
≥100	9	9.37 (3.55–24.74)	<0.001	83.4% (<0.001)
Study design				
Retrospective	22	9.23 (5.31–16.06)	<0.001	65.8% (<0.001)
Prospective	2	57.46 (5.18–637.15)	0.001	59.7% (0.115)
Country				
Asia	16	7.81 (4.15–14.68)	<0.001	65.7% (<0.001)
Non-Asia	8	18.26 (7.30–45.68)	<0.001	53.2% (0.037)
Cutoff				
≤1.18	11	10.16 (4.00–25.76)	<0.001	73.6% (<0.001)
>1.18	12	11.72 (5.12–26.83)	<0.001	72.0% (<0.001)
Time of CT measurement				
≤24 h	19	11.75 (5.84–23.66)	<0.001	75.8% (<0.001)
>24 h	5	10.25 (4.97–21.14)	<0.001	0% (0.555)
TTM				
All	12	5.96 (3.18–11.16)	<0.001	66.0% (0.001)
Part	6	17.79 (4.56–69.39)	<0.001	72.0% (0.003)
None	2	22.63 (2.82–181.78)	<0.001	0.0% (0.547)
Cardiac etiology				
≤50%	8	21.97 (10.86–44.46)	<0.001	0.0% (0.593)
>50%	9	4.51 (2.44–8.34)	<0.001	64.4% (0.004)
Row of CT				
16 rows	4	11.66 (2.22–11.66)	0.004	61.6% (0.050)
64 rows	5	8.76 (3.65–21.01)	<0.001	6.1% (0.372)

CI: confidence interval; CT: computed tomography; OR: odds ratio; TTM: targeted temperature management.

## Data Availability

The datasets used and/or analyzed during the current study are available from the corresponding author on reasonable request.

## Abbreviations

AUC: Area under the curve
CA: Cardiac arrest
CI: Confidence interval
GM: Gray matter
GWR: Gray-to-white ratio
OR: Odds ratio
TTM: Targeted temperature management
WM: White matter.

## Supplementary Materials

Supplementary materials Supplementary Fig 1: methodological quality summary.
