# Supplementary material for: Prognostic Values of the Gray-to-White Matter Ratio on Brain Computed Tomography Images for Neurological Outcomes after Cardiac Arrest: A Meta-Analysis
Source: Biomed Res Int. 2020 Nov 3;2020:7949516. doi: 10.1155/2020/7949516 (PMC7803139; doi:10.1155/2020/7949516)
Supplement: Supplementary materials — Supplementary Fig 1: methodological quality summary. [file 7949516.f1.pdf]

Supplementary Fig 1. Methodological quality summary.

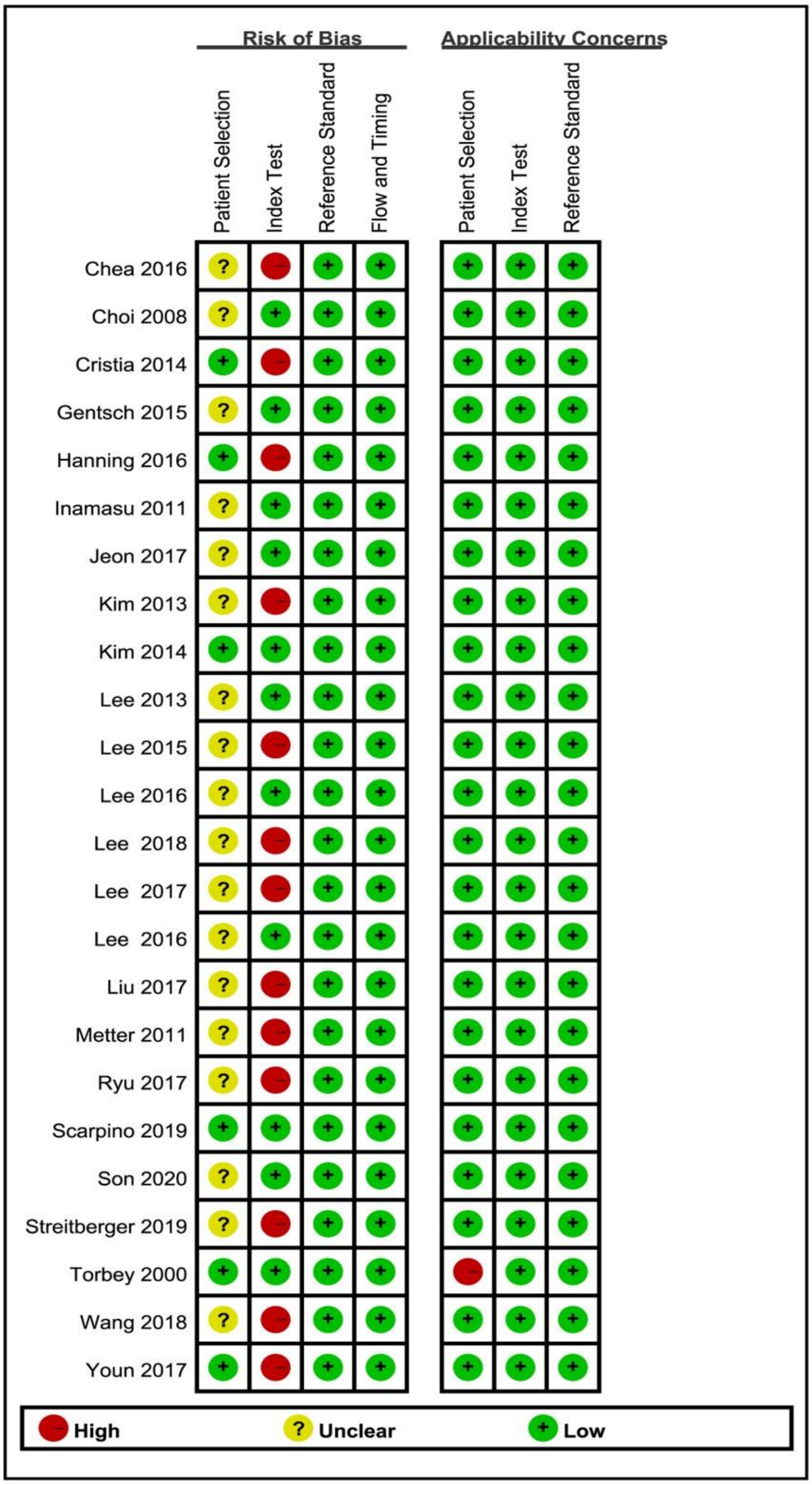
Risk of Bias
Applicability Concerns
Patient Selection
Index Test
Reference Standard
Flow and Timing
Patient Selection
Index Test
Reference Standard
Chea 2016
Choi 2008
Cristia 2014
Gentsch 2015
Hanning 2016
Inamasu 2011
Jeon 2017
Kim 2013
Kim 2014
Lee 2013
Lee 2015
Lee 2016
Lee 2018
Lee 2017
Lee 2016
Liu 2017
Metter 2011
Ryu 2017
Scarpino 2019
Son 2020
Streitberger 2019
Torbey 2000
Wang 2018
Youn 2017
High
Unclear
Low
